# Recent Advances in CRISPR/Cas Systems for Respiratory Pathogen Diagnostics

**DOI:** 10.3390/v18080870

**Published:** 2026-08-10

**Authors:** Yujie Dai, Lingyun Xia, Yufei Yang, Guohong Qiao, Xing Jin, Xuhua Mao

**Affiliations:** 1 Department of Clinical Laboratory, The Affiliated Yixing Clinical School of Medical School of Yangzhou University, Yixing 214200, China; yujiedai2404@163.com (Y.D.); yfyangyufei@163.com (Y.Y.); staff1000@yxph.com (G.Q.); 2 Department of Pediatrics, The Affiliated Yixing Clinical School of Medical School of Yangzhou University, Yixing 214200, China; staff575@yxph.com; 3 Department of Clinical Nutrition, The Affiliated Yixing Clinical School of Medical School of Yangzhou University, Yixing 214200, China

**Keywords:** CRISPR/Cas system, respiratory pathogens, molecular diagnostics

## Abstract

Early, rapid, and accurate detection is essential for clinical management and epidemiological control of acute respiratory infections caused by pathogens. Traditional testing methods such as microbial culture, serological testing, and PCR are restrictive in terms of operation and logistics and are therefore not easily used in point-of-care settings. The CRISPR/Cas system is an adaptive prokaryotic immune system composed of clustered regularly interspaced short palindromic repeats and their associated proteins, which has been used as a nucleic acid diagnostic platform with programmable sequence-specific target recognition and signal-amplifying collateral cleavage activity. Existing reviews have mostly focused on the classification of Cas enzymes or amplification strategies; in this review, a pathogen-centric approach was taken, covering viral pathogens (SARS-CoV-2, influenza virus, RSV, HAdV and VZV), bacterial pathogens (*Mycobacterium tuberculosis*, *Streptococcus pneumoniae*, *Mycoplasma pneumoniae* and *Staphylococcus aureus*) and fungal pathogens (*Aspergillus fumigatus* and *Pneumocystis jirovecii*). Key technological advances, such as isothermal amplification coupling, single-vessel integrated reaction designs, amplification-free digital detection, and electrochemical biosensor integration, are evaluated for Cas9-, Cas12-, and Cas13-based systems, with their mechanistic bases outlined. The current challenges that hinder clinical translation, such as sample matrix interference, multiple signal cross-talk, crRNA off-target effects, and the lack of large-scale validation studies, are critically assessed. At the same time, future pathways for portable, integrated, and inexpensive diagnostic platforms are suggested.

## 1. Introduction

Acute respiratory infections (ARIs) caused by viral, bacterial, and fungal pathogens are a significant burden on morbidity and mortality worldwide [1], and there is a need for reliable, rapid pathogen identification to enable optimal clinical care and epidemiological control. The current diagnostic methods that rely on microbial culture, serology, and polymerase chain reaction (PCR) have limitations. Culture-based methods may be ineffective for fastidious organisms and require a long incubation time [2]. Serological tests have a diagnostic window period during the early stage of infection and are prone to cross-reactivity, which can result in false-positive results. The high sensitivity and specificity of PCR are limited by the requirement for thermal cycling equipment and trained laboratory staff for its application in rapid, decentralized clinical settings [3] (Table 1). These limitations highlight the need for rapid, accurate, and flexible diagnostic platforms that are suitable for point-of-care and/or non-laboratory applications.

The CRISPR/Cas system has been developed into a nucleic acid detection system based on programmed sequence-specific target recognition and collateral target cleavage activity [4]. Its compatibility with isothermal amplification techniques, such as loop-mediated isothermal amplification (LAMP), recombinase polymerase amplification (RPA), and enzymatic recombinase amplification (ERA), has enabled its sensitivity to reach the attomolar (aM) and zeptomolar (zM) range [5]. Amplification-coupled CRISPR/Cas platforms have several practical drawbacks, such as complex primer and crRNA designs, higher reagent costs, and the potential for false-positive results due to aerosol contamination [6,7]. To overcome these challenges, amplification-free CRISPR/Cas-based detection systems and one-step assay formats have attracted increasing research attention. In addition to nucleic acid detection, CRISPR/Cas technologies have also been modified for other non-nucleic acid (NNA) targets such as proteins [8], antigen–antibody complexes [9], and small metabolic molecules [10,11], and can be further adapted for rapid diagnostic applications for respiratory infectious diseases.

**Figure 1:**
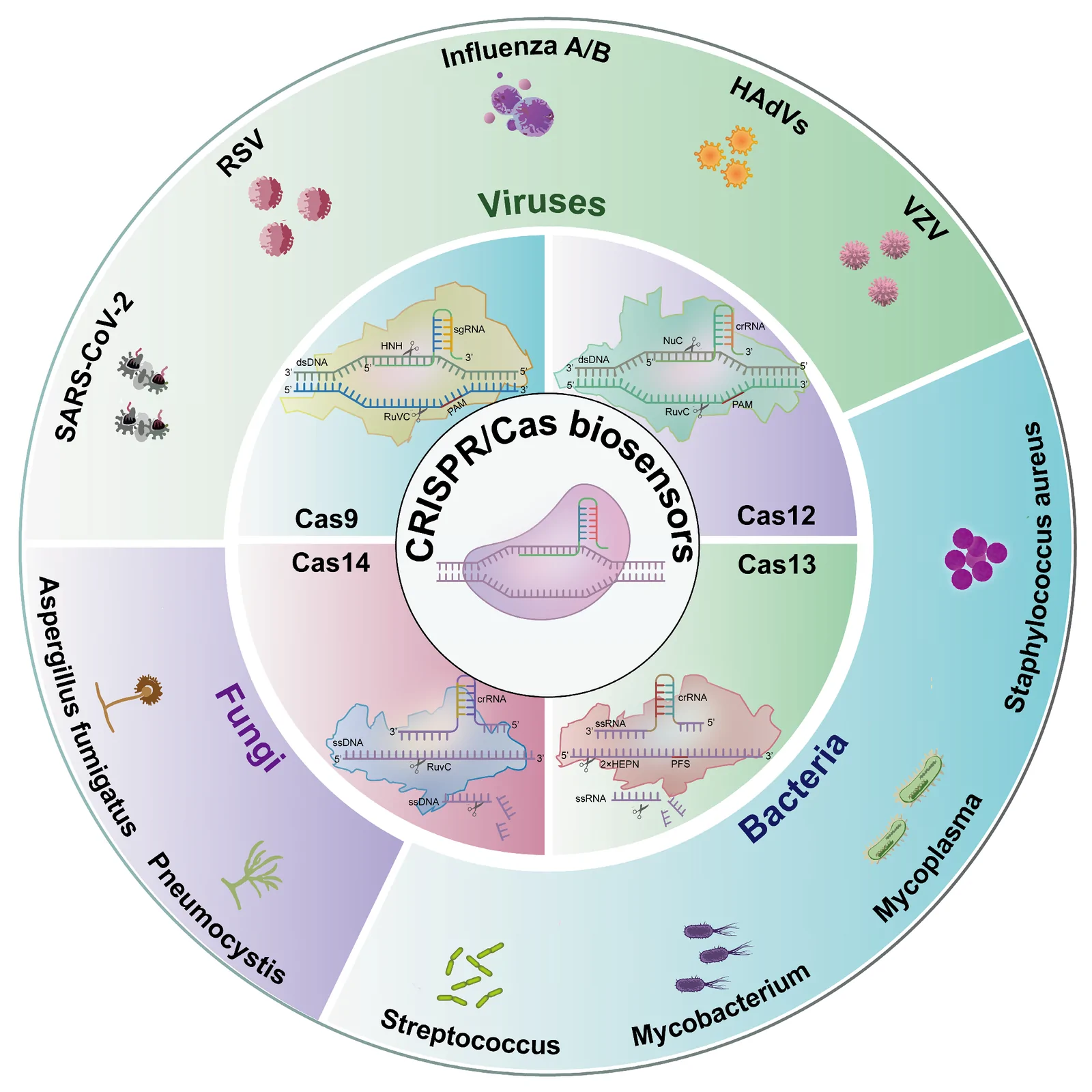
Schematic representation of CRISPR/Cas-based biosensors for respiratory pathogen detection.

Most current CRISPR/Cas diagnostics reviews are centered on multidrug-resistant clinical bacteria, foodborne pathogens, or RNA viruses and have been classified according to detection technologies or Cas effector proteins. Few studies, however, have explored the use of CRISPR/Cas for the detection of respiratory pathogens from a clinical epidemiology perspective. In this review, we introduce the different types of CRISPR/Cas-based diagnostic systems and the mechanism of Cas9, Cas12, and Cas13-mediated detection (Figure 1); discuss recent technological developments and challenges; and highlight the potential applications of CRISPR/Cas-based detection for viral, bacterial, and fungal respiratory pathogens, creating a consolidated framework for further research in the field of CRISPR-based detection.

**Table 1:** Comparison of four molecular diagnostic methods.

Method	Time Required	Cost (per Test)	Advantages	Limitations
Microbial culture	3–5 days	Low (USD ~13–50/test)	Suitable for many sample types; allows drug susceptibility testing	Time-consuming; high false-negative rate for fastidious bacteria
Serology	1–2 h	Low (USD ~20–80/test)	No specialized equipment required; suitable for mass screening	Window period; cross-reactivity
qRT-PCR	1–2 h	Higher cost of equipment and reagents	Gold standard; high sensitivity and specificity	Skilled personnel and specialized laboratory
CRISPR/Cas system	0.5–1 h	Low to medium for on-site inspection (<USD 6.5/test) [12]	Low cost; high sensitivity; minimal equipment; suitable for point-of-care testing	Lacks large-scale clinical validation

## 2. Classification and Mechanism of the CRISPR/Cas System

The CRISPR/Cas system can be divided into two major classes: the Class I system, which utilizes a multi-Cas protein complex, and the Class II system, which uses a single-Cas effector protein. The Classes I and II comprise types I, III, IV, V, and VI (Cas9, Cas12, and Cas13), respectively [13]. The Class II system is structurally simpler, requiring only one Cas protein to form a functional ribonucleoprotein complex with a crRNA. Precise target recognition and cleavage are achieved through sequence-specific crRNA design. When Cas proteins are activated, they can also cleave nearby single-stranded nucleic acids, producing detectable signals for diagnostic applications, a phenomenon known as collateral (non-specific) cleavage. This simplicity has enabled the widespread use of the Class II system for gene editing and molecular diagnostics [14]. Cas9, Cas12, and Cas13 are the most popular diagnostic effector proteins in Class II systems. Cas9 primarily targets double-stranded DNA (dsDNA) and functions through a ribonucleoprotein (RNP) complex formed with a single-guide RNA (sgRNA), which is a fusion of CRISPR RNA (crRNA) and trans-activating crRNA (tracrRNA). The sgRNA directs Cas9 to recognize target DNA sequences adjacent to a protospacer adjacent motif (PAM), after which the RuvC and HNH nuclease domains introduce a double-strand break (DSB), enabling gene editing [15,16]. Although Cas9 lacks collateral cleavage activity, its high sequence specificity makes it valuable for diagnostic applications that rely on target recognition. The Cas12 system (e.g., Cas12a, Cas12b) exhibits a cleavage mechanism distinct from that of Cas9. Guided by crRNA, Cas12 recognizes target dsDNA and mediates site-specific cleavage via its RuvC domain, referred to as cis-cleavage activity [17]. Following target recognition and activation, Cas12a acquires non-specific trans-cleavage activity against single-stranded DNA (ssDNA) [18]. This collateral cleavage activity underlies its extensive application in molecular diagnostics. Cas13a is an RNA-guided endoribonuclease (endoRNase) that specifically cleaves single-stranded RNA (ssRNA) [19]. Like Cas12, it exhibits collateral cleavage activity upon activation, making it well-suited for the detection of RNA viruses. Cas14 is a microRNA-guided nuclease that prefers ssDNA and does not require a PAM sequence, which is another option for nucleic acid detection [20]. The properties and differences of popular Class II Cas effector proteins have been summarized in Table 2. Table 3 further compares these Cas systems in the context of respiratory pathogen detection.

**Figure 2:**
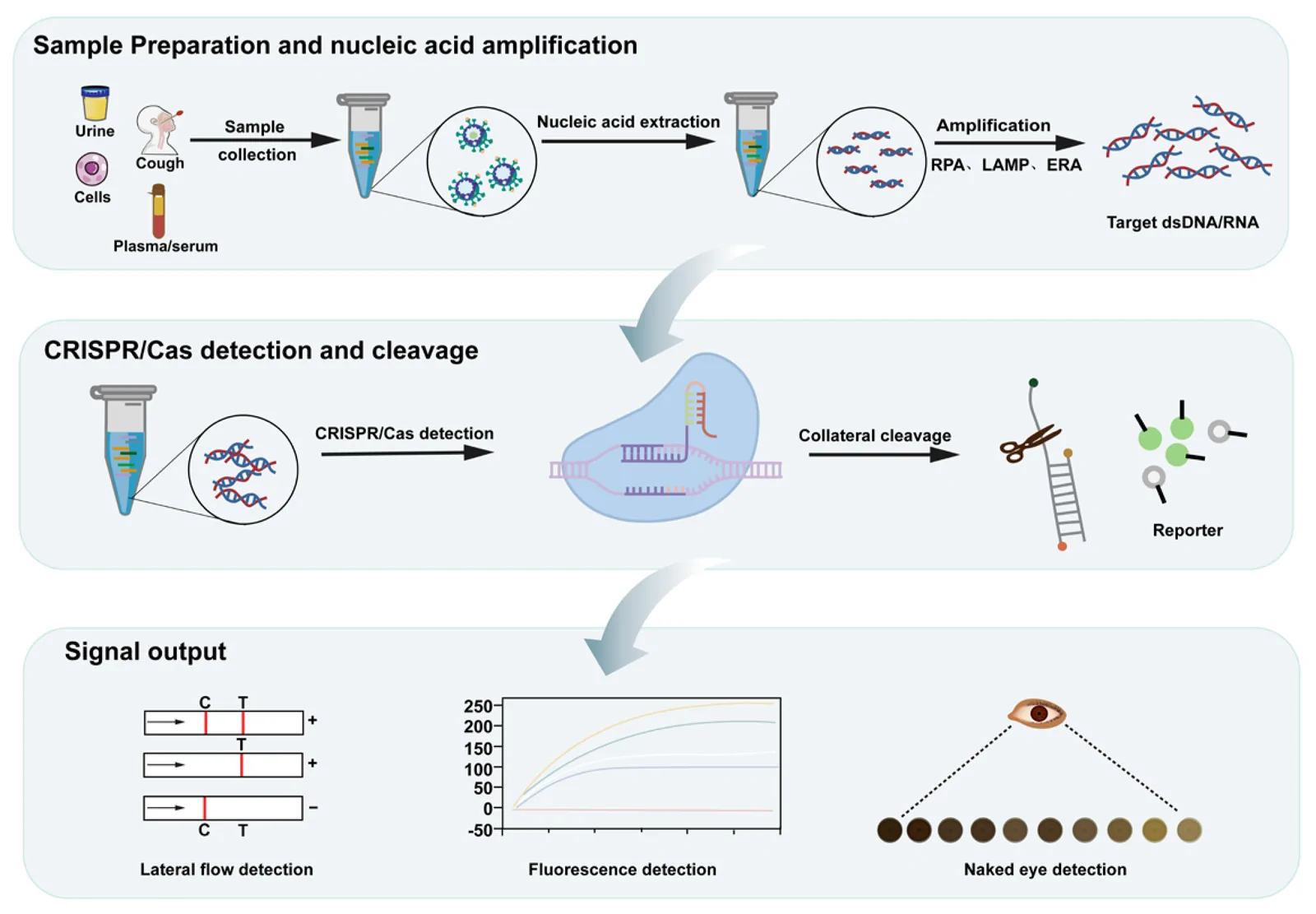
Workflow of CRISPR/Cas-based nucleic acid detection for pathogens.

Typical diagnostic workflows with CRISPR-Cas involve three sequential steps [21]: nucleic acid extraction and amplification to increase sensitivity; recognition and activation by CRISPR/Cas, which includes target nucleic acids binding to the Cas protein and binding of the specific gRNA/crRNA to activate the nuclease activity; and signal reporting, which involves the target nucleic acids being recognized by the activated CRISPR/Cas complex and cutting pre-added reporter molecules containing fluorescent, colorimetric, or electrochemical tags to generate outputs that can be detected by fluorometers, by visual inspection, or via electrochemical detection platforms (Figure 2).

## 3. Application of the CRISPR/Cas System in Respiratory Pathogen Detection

As detection platforms using Cas9, Cas12, and Cas13 improve, CRISPR/Cas technology has been widely used for the detection of various respiratory pathogens, including viruses, bacteria, and fungi. These approaches provide benefits in terms of diagnostic sensitivity, speed, and accuracy. Below, we highlight existing uses of CRISPR/Cas systems for detecting respiratory pathogens.

### 3.1. Viral Respiratory Pathogens

#### 3.1.1. Severe Acute Respiratory Syndrome Coronavirus 2 (SARS-CoV-2)

SARS-CoV-2 is a positive-sense single-stranded RNA virus. It has four structural proteins encoded in its genome: Nucleocapsid (N), Membrane (M), Spike (S), and Envelope (E). Of these, the S and N proteins are usually used as targets for SARS-CoV-2 detection [22]. Currently, real-time quantitative RT-PCR (qRT-PCR) is considered the “gold standard” for detecting SARS-CoV-2 [23,24]. In practice, however, there are some limitations, and this technique cannot be used for rapid point-of-care screening or mass testing [25]. The CRISPR/Cas diagnostic systems offer greater specificity and sensitivity and have emerged as a key tool for detecting SARS-CoV-2.

To detect SARS-CoV-2, several studies have combined CRISPR/Cas systems with isothermal amplification methods, yielding a highly sensitive nucleic acid detection strategy. LAMP was combined with Cas12-based detection to allow for simultaneous reverse transcription and isothermal amplification of RNA from nasopharyngeal and oropharyngeal swabs [26]. This approach aimed to target the *N* and *E* genes of SARS-CoV-2, as well as the human RNase P gene, which serves as an internal control. Either lateral flow strips or fluorescence readouts were used to detect the signals. It had a positive percent agreement of 95% and a negative percent agreement of 100%, compared with qRT-PCR, which took about 40 min to obtain results. Because of isothermal amplification, the assay is fast, accurate, and can be easily read out in a variety of formats. It however has some drawbacks in common with qRT-PCR: it requires a relatively complex workflow, is reliant on specialized equipment and reagents, and may suffer from off-target amplification.

**Figure 3:**
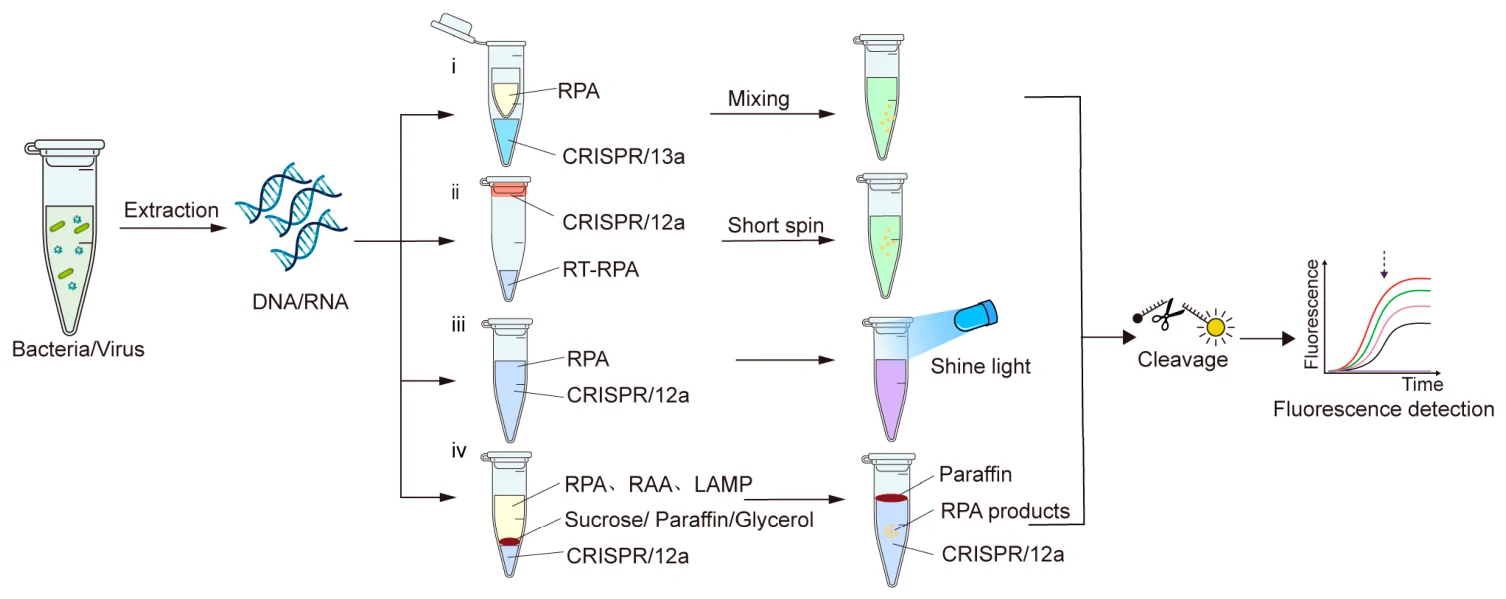
Schematic illustration of one-step amplification-based CRISPR/Cas detection platforms.

To address these challenges, integrated systems have been developed that integrate amplification and CRISPR reactions into a single workflow. All these methods rely on physical separation techniques or optimized reaction conditions to achieve one-pot detection (Figure 3). Hu et al. [27] constructed a double-layer physical separation reaction tube for the one-pot detection of SARS-CoV-2. The RPA reaction was carried out in the inner chamber, which was equipped with two hydrophobic pores at its bottom. The reaction was then amplified and transferred to the outer chamber by centrifugation or shaking, where the pre-stored Cas13a reagent was present, which allowed nucleic acid detection without opening the tube (Figure 3). The assay had a detection limit of 3 copies/µL and was comparable to qRT-PCR for both positive and negative samples. This method did not require any special instrumentation, reduced assay time, and lowered the overall assay cost. In the same way, Sun et al. [28] loaded the CRISPR reaction mixture onto the cap of the reaction tube, and the RT-RPA mixture was loaded onto the bottom of the tube. Both were amplified and then mixed by centrifugation (Figure 3). This system enabled SARS-CoV-2 detection within 50 min, with a limit of detection of 1 copy/μL. Hu et al. [29] Further developed a photocontrolled CRISPR/Cas12a detection platform for SARS-CoV-2. In this design, the RPA and CRISPR/Cas12a components were separated within a sealed tube, with crRNA maintained in an inactive state to prevent premature Cas12a activation during amplification. After completion of RPA, the CRISPR/Cas12a system was activated by light, and results were interpreted using a smartphone-based application (Figure 3). Compared with qRT-qPCR, this method demonstrated 90% sensitivity and 100% specificity. Compared with conventional one-pot systems, it eliminated the need for manual intermediate steps such as centrifugation or mixing, reducing contamination risk and improving suitability for automated or point-of-care testing (POCT).

**Figure 4:**
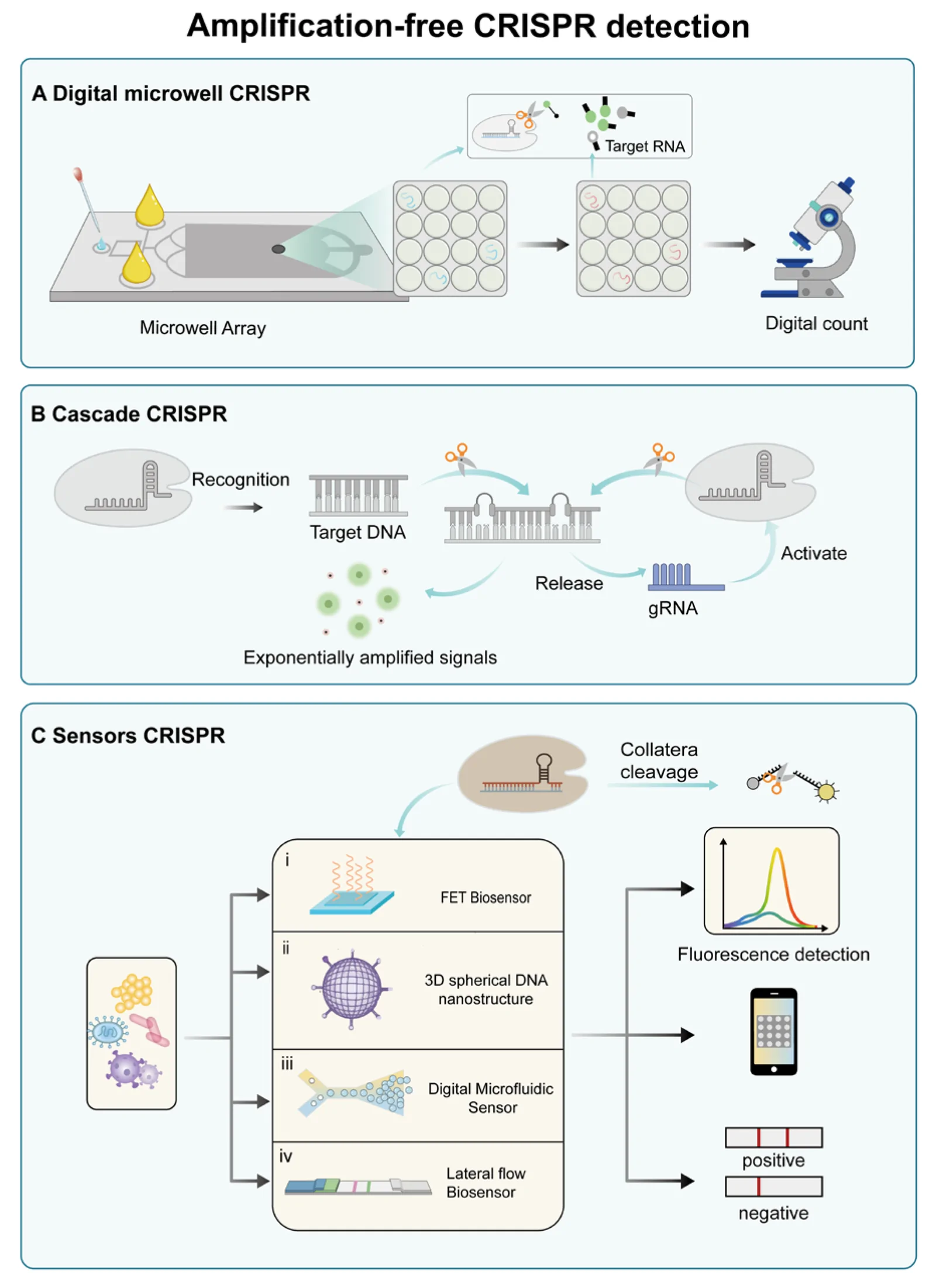
Schematic representation of key amplification-free CRISPR-based detection strategies.

Wang et al. [30] developed an integrated detection platform combining CRISPR/Cas13a with nucleic acid enrichment in microwell arrays and single-molecule digital detection. Magnetic beads captured and concentrated targets from large samples, and the target-triggered CRISPR/Cas13a cleavage reaction was compartmentalized into millions of femtoliter-sized microwells (Figure 4A). This enhanced local signal intensity enables a “sample-in, answer-out” amplification-free single-molecule detection of SARS-CoV-2. It significantly improved sensitivity and further mitigated two major issues associated with traditional methods: amplification bias and aerosol contamination.

#### 3.1.2. Influenza Virus

Influenza viruses are major pathogens responsible for global pandemics and seasonal influenza. They are classified into types A, B, C, and D [31]. According to WHO estimates, influenza causes 3–5 million severe cases and approximately 290,000–650,000 respiratory disease-related deaths annually worldwide [32]. Rapid identification of influenza viruses is therefore essential for effective outbreak prevention and control. Zhou et al. [33] developed a colorimetric biosensor for detecting H1N1 influenza virus based on the CRISPR/Cas13a system combined with a hybridization chain reaction (HCR). In this system, Cas13a-mediated cleavage of probe RNA triggers the HCR cascade, resulting in a visible green colorimetric signal. The detection limit of the assay was 0.152 pM. Sensitivity, specificity, and visual readout are improved by signal amplification via the chain reaction. There are some drawbacks, though, such as the relatively high cost, the fact that the Cas proteins can act off-target, and the potential for false-positive results if the system is contaminated. Zhang et al. [34] designed a nucleic acid-free fluorescent biosensor based on a three-dimensional spherical DNA nanostructure (3D-SDNC) integrated with the CRISPR/Cas12a system for rapid and ultrasensitive detection of H1N1 influenza virus. The biosensor was constructed by assembling six ssDNA molecules into a 3D-SDNC framework, with each vertex functionalized with H1N1-specific aptamers and an activation strand for Cas12a. In the presence of the H1N1 virus, aptamer-virus binding induces a conformational change that activates the Cas12a/crRNA complex, leading to trans-cleavage of fluorophore-labeled reporter probes and generation of an amplified fluorescence signal (Figure 4C). The viral presence was ultimately determined by measuring fluorescence intensity. This study integrates DNA nanotechnology with CRISPR-based sensing to establish a novel diagnostic platform with strong potential for early influenza detection and for developing more stable biosensors.

In addition, influenza virus sequence data have taken on heightened importance in the context of increasing circulation of drug-resistant strains. Oseltamivir, a neuraminidase inhibitor, remains the most widely prescribed antiviral agent for seasonal influenza [35]. Nevertheless, a single nucleotide polymorphism (SNP) in the neuraminidase gene leads to the H275Y amino acid substitution, which confers oseltamivir resistance in H1N1 influenza A virus [36]. To address this challenge, Zhang et al. [37] developed the SHINE platform based on a dual Cas12a/Cas13a detection system. This assay is capable of discriminating between the two alleles of the H275Y resistance mutation, thereby enabling rapid identification of oseltamivir-resistant strains. The platform thus provides a novel tool for antiviral resistance surveillance in influenza viruses and may assist clinicians in making more informed therapeutic decisions at the point of care.

The H5N1 highly pathogenic avian influenza (HPAI) virus was first detected in poultry in Guangdong Province, China, in 1996 [38]. Since its emergence, the virus has progressively spread across Asia, Europe, Africa, and even Antarctica, driving cross-species transmission and regional epidemics [39]. By 2025, epidemiological data indicated that the mortality rate attributed to H5N1 infection had surpassed 50% [40]. In this context, the development of rapid and accurate detection methods carries substantial public health importance for early case identification and outbreak containment.The CRISPR/Cas-based lateral flow assay (LFA) has emerged as a promising approach in this regard. Li et al. [41] integrated recombinase-mediated nucleic acid amplification with the CRISPR-Cas13a system and LFA technology, achieving highly sensitive detection of H5 subtype avian influenza virus in clinical specimens. The assay demonstrated a limit of detection of 0.1 copies/μL, showed complete concordance with qRT-PCR results, and exhibited no cross-reactivity with other subtypes, including H3, H7, H9, and H10.Building upon these findings, Guo et al. [42] constructed a CRISPR/Cas12a-based multimodal biosensing platform equipped with a triple-signal readout capability. This platform enables detection of the H5N1 virus within one hour, with a limit of detection of 3.7 × 10^2^ copies/μL, and supports three independent signal output modes—fluorescence, lateral flow strips, and portable glucose meters—thereby offering enhanced flexibility for deployment in resource-constrained environments. The inherent specificity of the CRISPR system, combined with the operational simplicity of LFA, renders this strategy particularly well-suited for point-of-care testing applications. Although CRISPR-based HPAI-LFA products have yet to reach the commercial stage, ongoing translational research continues to advance the practical implementation of this technology.

#### 3.1.3. Respiratory Syncytial Virus (RSV)

RSV is a leading cause of severe lower respiratory tract infections in infants, elderly individuals, and immunocompromised populations [43,44]. CRISPR/Cas systems have also demonstrated strong performance in RSV detection. In recent years, integration of RPA with CRISPR/Cas platforms has enabled the development of rapid and highly specific RSV diagnostic systems [45,46].

Tan et al. [47] developed a “one-pot RPA-CRISPR/Cas12a” system enabling simultaneous detection of 12 common respiratory pathogens, including SARS-CoV-2, influenza virus, and RSV. In this design, paraffin was used to physically separate the RPA and CRISPR/Cas12a components, ensuring sufficient amplification of target sequences before CRISPR activation (Figure 3). This system highlights the potential of CRISPR/Cas platforms for multiplex pathogen detection and has important implications for distinguishing bacterial and viral infections to support rational antibiotic use. The one-pot format reduces the risk of aerosol contamination. However, despite its advantages, pathogen identification in this system still depends on pre-aliquoted reagents or repeated testing, and true single-tube multiplex signal discrimination remains to be further optimized. Wang et al. [48] developed a self-cascading cyclic amplification method (SCC-Cas13a) based on LbuCas13a, in which a hairpin probe containing multiple uracil residues was designed. Upon target RNA-activated Cas13a cleavage of the probe, RNA activators identical to the target sequence are released, triggering a new round of CRISPR activation and establishing a positive feedback loop that enables exponential signal amplification without the need for pre-amplification(Figure 4B). This method enables simultaneous detection of RSV, influenza A virus, and SARS-CoV-2 within 3 min. Integrated with a smartphone-assisted device, the system achieved multiplexed point-of-care testing of respiratory viruses in clinical nasopharyngeal swab samples, providing a powerful tool for rapid respiratory virus screening in resource-limited settings. Li et al. [49] Developed an amplification-free nucleic acid detection platform using Cas13a-integrated graphene field-effect transistors (gFETs), enabling direct detection of SARS-CoV-2 and RSV genomes without prior amplification (Figure 4C). In this system, Cas13a recognition of target RNA activates non-specific cleavage of RNA reporter molecules immobilized on the graphene surface, resulting in measurable electrical signal changes in the gFET. This approach achieves a detection limit of 1 AM and significantly improves assay speed and sensitivity while reducing overall assay time.

#### 3.1.4. Human Adenoviruses (HAdVs)

HAdVs are common causes of respiratory infections in children, accounting for approximately 5–10% of lower respiratory tract infections in this population. They are also associated with multisystem infections, including respiratory, ocular, and gastrointestinal diseases [50]. Gao et al. [46] combined RPA with a CRISPR/Cas detection system and designed specific primers and crRNAs based on multiple sequence alignments to enable dual detection of RSV and HAdVs. The assay was 98.1% sensitive for RSV and 91.4% sensitive for HAdV in 160 clinical throat nasopharyngeal swab samples, and had 100% specificity for both compared with qPCR. The dual-target amplification strategy helps to streamline workflow and enhance detection efficiency. However, interactions between the primer and the crRNA remain an important challenge for the development of multiplex assays. A detection method for HAdV55 was developed by Zhang et al. [51] based on enzymatically recombinase amplification (ERA) of the HAdV55 gene, with CRISPR/Cas12a-mediated signal enhancement targeting the conserved region of the hexon gene. The system has been validated using plasmid standards and different dilutions of cultured strains, with optimized primers and crRNAs, to evaluate analytical sensitivity. This approach has high sensitivity and specificity, does not require complex instrumentation, and can be used for fast screening at the point of care.

#### 3.1.5. Varicella-Zoster Virus (VZV)

The natural reservoir for VZV is humans, and the infection rate is >90% worldwide [52], It is responsible for the disease chickenpox during the initial infection. It can reactivate later in life, especially in the elderly and in patients with a weakened immune system, causing the disease shingles. There can also be severe complications, such as meningitis and encephalitis [53]. Zhong et al. [54] used RPA to amplify the VZV gE gene and designed a lateral flow immunochromatographic assay for detection, which used quantum dot nanobead (QDNB) labeling. The outcomes could be visualized within 1 h, and the evaluation of 86 clinical vesicle samples showed 100% correlation with qPCR results. The Cas12a-QDNBs system has the advantage of using minimal sample volume and can accurately detect without nucleic acid extraction, making it suitable for rapid screening in resource-constrained areas.

### 3.2. Bacterial Respiratory Pathogens

#### 3.2.1. *Mycobacterium tuberculosis* (MTB)

*Pulmonary tuberculosis* is caused by only one organism, *Mycobacterium tuberculosis*. Worldwide, there are about 10 million new cases and about 1.5 million deaths every year. WHO estimates that 8.2 million new cases of TB occurred worldwide in 2023 [55]. Gan et al. [56] developed an ERA-CRISPR/Cas12a detection system for fast identification of MTB. The assay was 100% specific, as it did not cross-react with other respiratory pathogens or non-tuberculous mycobacteria. Cas12a is used to target MTB DNA, with both fluorescence and lateral flow readouts. The dual-mode format enables high sensitivity in the laboratory and fast point-of-care screening. The two-step protocol, however, still involves the manual transfer of amplified products, which adds complexity to the procedure and can lead to contamination. Peng et al. [57] constructed a one-step tuberculosis detection system that integrates CRISPR/Cas12b and cross-priming amplification (CPA) for the detection of tuberculosis in sputum samples, which enhances the diagnostic efficiency and streamlines the workflow. This makes nucleic acid amplification and CRISPR detection of sequences easier, minimizing the risk of cross-contamination. The assay had a detection limit of 0.8 copies/μL and a sensitivity of 67.2% when used with H37Rv genomic DNA. There was no cross-reaction with non-tuberculous mycobacteria or other respiratory pathogens, and specificity was 96.7%. The use of Cas12b further enhances robustness and detection efficiency.

Liao et al. [58] addressed the need for accessible and low-cost diagnostic solutions with the development of the dAFCas-TB platform, which combines heat-based lysis, microdroplet digital CRISPR, and smartphone-assisted imaging. Clinical evaluation of 58 MTB samples showed a sensitivity of 97.7% and a specificity of 93.8%. This system is well-suited for large-scale screening in resource-limited settings. It can contribute to the development of rapid, accurate, and cost-effective CRISPR-based diagnostics for tuberculosis at a cost of less than USD 1 per test.

The increasing prevalence of multidrug-resistant tuberculosis (MDR-TB) has created an urgent demand for accurate and specific diagnostic tools capable of identifying Mycobacterium tuberculosis complex (MTBC) strains resistant to rifampin (RFP) and isoniazid (INH), with the goal of curbing further transmission. Xiao et al. [59] constructed a CRISPR/Cas14a-based platform for the simultaneous detection of RFP and INH resistance in MTB. This system co-amplifies the rpoB, katG, and inhA genes in a single multiplex reaction, and the amplicons are then transferred to separate chambers preloaded with Cas14a, guide RNAs, T7 exonuclease, and a fluorescent reporter. Results are read using a fluorescence microplate reader. Two guide RNAs—gRNA-WT and gRNA-M were designed to specifically recognize wild-type and mutant alleles, respectively, and resistance status was determined by fluorescence intensity. In validation experiments using 60 clinical isolates, the assay demonstrated 93.3% sensitivity and 100% specificity for RFP resistance detection, and 97.5% sensitivity with 100% specificity for INH resistance detection.

#### 3.2.2. *Streptococcus pneumoniae* (SP)

*Streptococcus pneumoniae* is a significant pathogen that causes community-acquired pneumonia, meningitis, and otitis media. Zhou et al. [60] combined LAMP and Cas12a collateral cleavage activity to develop a lateral flow immunochromatographic platform for the detection of the cpsA gene of SP. The system was used to detect SP in clinical respiratory samples and influenza A virus. The detection was performed within 35 min. Feasibility and diagnostic performance were shown in 23 bronchoalveolar lavage and nasopharyngeal swab samples. After nucleic acid extraction, samples were amplified by LAMP and then detected using the CRISPR/Cas12a fluorescence detection system, with a total assay time of less than 1 h, making it suitable for rapid on-site testing. Kan et al. [61] developed a single-tube, palm-sized CRISPR/Cas12a diagnostic platform (PaCD) to enhance sensitivity further, streamline workflows, reduce contamination risk, and increase multiplexing capacity. This system allows the detection of all four pathogens, namely SP, *Chlamydia pneumoniae*, *Haemophilus influenzae*, and *Mycoplasma pneumoniae*, in one reaction vessel at the same time. Combined with a portable, temperature-controlled readout device, the entire diagnostic workflow takes just 30 min, increasing the feasibility of point-of-care testing. The PaCD system showed 100% agreement with next-generation sequencing results, demonstrating its clinical utility for multiplex respiratory pathogen detection.

#### 3.2.3. *Mycoplasma pneumoniae* (MP)

*Mycoplasma pneumoniae* is one of the most important etiological agents of upper respiratory tract infections in children, adolescents, and adults, which usually manifest as a dry cough, fever, and chest discomfort [62]. Deng et al. [63] integrated CRISPR/Cas12a with ERA to create a dual-mode detection platform for MP, with both fluorescence and lateral flow modes. The assay results are available in about 30 min. The system achieved 100% specificity and a 1-copy/µL detection limit in a clinical evaluation of 92 samples. In this study, the first successful combination of ERA and CRISPR/Cas12a for MP detection was proposed, showing the potential of isothermal amplification-CRISPR hybrid strategies for rapid nucleic acid diagnostics. Following this idea, Nan et al. [64] designed an MP-MCDA-CRISPR system, which is a combination of multiple cross-displacement amplification (MCDA) and CRISPR/Cas12a detection. The whole process takes less than 1 h and combines the sample lysis, MCDA, and CRISPR detection. Validation with 128 clinical samples achieved 50 correct positive and 78 correct negative results for qPCR-positive and qPCR-negative samples, respectively. This system also exhibits enhanced clinical applicability and provides further progress in the translation of isothermal amplification-CRISPR systems for MP diagnostics.

Macrolide antibiotics remain the cornerstone of treatment for Mycoplasma pneumoniae (MP) pneumonia in pediatric patients. In recent years, however, the growing prevalence of macrolide resistance has emerged as a major contributor to refractory pneumonia [65]. MP strains harboring the A2063G mutation in the 23S rRNA gene are of particular concern, as they have been closely associated with severe disease manifestations, increased complication rates, and long-term sequelae [66]. Therefore, a sensitive, rapid, and reliable diagnostic approach capable of both MP detection and A2063G mutation identification is urgently needed to support early differential diagnosis and inform appropriate antibiotic selection. Building on their previously established one-step RPA-CRISPR/Cas13a fluorescence assay, Ren et al. [67] developed a two-step detection system specifically designed to identify the A2063G macrolide resistance mutation, achieving a limit of detection of 10 copies/μL. When tested on 27 clinical specimens, the assay showed complete concordance with sequencing data, thereby validating the utility of this RPA-CRISPR/Cas13a-based platform for simultaneous detection of MP nucleic acid and the A2063G resistance mutation.

#### 3.2.4. *Staphylococcus aureus*

*Staphylococcus aureus* is a frequent cause of community-acquired pneumonia, hospital-acquired pneumonia, and respiratory co-infection. The introduction of methicillin-resistant *Staphylococcus aureus* (MRSA) has added to the clinical challenge. Xu et al. [68] constructed an RPA-Cas12a fluorescence detection system, which was specific to the *gE* gene of *S. aureus*. The assay yields isothermal amplification in just 30 min, and a fluorescence signal is generated by Cas12a-crRNA-mediated cleavage of FAM-labeled reporter probes. The detection limit was 10 copies per reaction, and there was no cross-reactivity with common respiratory pathogens, suggesting strong potential for rapid screening of respiratory infections. A modular microfluidic sensing platform for RNA detection was designed by Mao et al. [69] which combines nucleic acid extraction, CRISPR/Cas13a, and electrochemiluminescence (ECL) readout. A surface-tension-driven filtration strategy is used to accelerate sample processing, and a multi-channel CRISPR/Cas13a system is used for simultaneous target recognition and signal amplification. A closed bipolar electrode ECL configuration also simplifies operation and increases analytical sensitivity. This all-in-one platform can perform quick RNA multiplex detection with *Escherichia coli* and *S. aureus* as model targets.

### 3.3. Fungi Respiratory Pathogens

#### 3.3.1. *Aspergillus fumigatus*

*Aspergillus fumigatus* is a filamentous saprophytic fungus that is present in soil and places of frequent human contact. Has a high tolerance to environmental stress and persistence. In persons with compromised immune systems, inhaled spores can persist and multiply in host tissue, causing various forms of invasive aspergillosis, including pulmonary aspergillosis, meningitis, and endocarditis, which can be fatal in extreme cases [70]. Conventional fungal culture techniques are time-consuming and less sensitive, which limits their usefulness for early diagnosis of fungal infections. Lin et al. [71] developed a diagnostic method based on RPA-CRISPR/Cas12a to detect *Aspergillus fumigatus* in the lungs by targeting the β-tubulin gene. This system combines isothermal amplification and single-strand DNA signal generation using Cas12a. To reduce aerosol contamination during amplification, liquid paraffin is placed on top of the reaction mixture to prevent aerosol exposure during tube opening. Detection is then read out on a fluorescence or lateral flow strip. The validation with 49 clinical samples (qPCR as the reference) showed 100% specificity and a limit of detection of 10^2^ copies/μL, overcoming the limitations of conventional assays, including low detection limits and long turnaround times. One problem that remains is that it may not discern between colonization, contamination, and true invasive infection. In 2022, WHO added *Aspergillus fumigatus* to the list of high-priority fungal pathogens [72]. largely because of its significant burden of disease and growing antifungal resistance. The TR34/L98H mutation in cyp51A is a well-described mechanism of azole resistance, of particular concern [73]. This mutation confers cross-resistance to the most widely used antifungal drugs, making treatment strategies more difficult. The future could also involve integrated resistance and pathogen profiling using CRISPR-based multi-target detection strategies, as knowledge of resistance mechanisms continues to develop.

#### 3.3.2. *Pneumocystis jirovecii*

*Pneumocystis jirovecii* is the organism responsible for Pneumocystis pneumonia (PJP), a disease seen mainly in persons with impaired immune systems. It is still a significant opportunistic pathogen related to morbidity and mortality, especially in acquired immunodeficiency syndrome (AIDS) patients [74]. WU et al. [75] developed a recombinase polymerase amplification (RPA)-CRISPR/Cas12a system targeting a conserved region of the mitochondrial mtSSU rRNA gene in *Pneumocystis jirovecii*. The diagnostic signal is produced by Cas12a-mediated cleavage of reporter probes after target amplification. No cross-reactivity was observed with 15 other common respiratory pathogens, including *Aspergillus fumigatus*, *Aspergillus flavus*, *Aspergillus niger*, and *Mycobacterium tuberculosis*. The system offers a quick and accurate diagnostic method for PJP. Still, it has been evaluated in a relatively small population at a single center and cannot differentiate between active infection and colonization. Thus, larger multi-center studies are needed for more robust validation of clinical performance. Ulloa et al. [76] developed a platform for the detection of CRISPR-Cas12a that does not require nucleic acid amplification and can be read out in either fluorescence or lateral flow. The assay targets DHPS, β-tubulin, and mtLSU rRNA genes, including the β-tubulin-targeting crRNA, which provides a detectable fluorescence signal within 20–30 min. The system is easy to use, inexpensive, and eliminates the need for thermal cycling equipment, making it ideal for point-of-care use in resource-limited settings.

## 4. Conclusions and Outlook

Given their specificity and programmability, CRISPR/Cas systems have become a highly versatile tool for detecting a wide range of respiratory pathogens. They are used in conjunction with diagnostic tools such as SHERLOCK, DETECTR, and SHINE to enable fast, accurate identification of pathogens. Moreover, the combination of CRISPR/Cas systems with microfluidic devices, electrochemical biosensors, and even DNA nanomachines has led to highly sensitive, highly specific, and even amplification-free detection approaches. These developments also aid multiplexed detection of various pathogens and markers of antimicrobial resistance [77].

However, some obstacles remain that hinder the widespread clinical use of CRISPR-based respiratory diagnostics. (1) Complex sample processing: There are systems available that offer “sample-in, result-out” capabilities. Nasopharyngeal and oropharyngeal swabs have largely become the primary sample types in CRISPR/Cas-based respiratory pathogen detection, owing to their ease of collection, favorable patient tolerance, and relatively high viral loads during the acute phase of respiratory viral infections. Bronchoalveolar lavage fluid (BALF), although regarded as the reference standard for diagnosing lower respiratory tract infections, requires an invasive bronchoscopic procedure, which limits its feasibility for large-scale population screening. Sputum, frequently used for tuberculosis detection, contains abundant mucus, host genomic DNA, and various enzymatic inhibitors that may compromise CRISPR/Cas assay performance and potentially contribute to false-positive or false-negative readouts [78]. Other biological specimens, including urine, saliva, and whole blood, are less commonly employed. Several factors account for this: the pathogen burden in non-respiratory matrices tends to be substantially lower; blood-derived proteins and heme can interfere with Cas enzyme activity [79]; the degree of inhibition on isothermal amplification and CRISPR reactions varies considerably across different specimen types; and standardized sample pretreatment protocols remain largely unavailable [80]. Today, most workflows still rely on time-consuming, costly steps involving nucleic acid extraction. To overcome this, simplified and automated sample processing methods, such as extraction-free methods [81], one-pot reactions, thermal lysis, and microfluidic integration, have been investigated to simplify workflows and minimize contamination [82]. (2) Off-target and background effects: The non-specific collateral cleavage activity of Cas proteins is adopted as a signal amplification mechanism but can also result in background signals or off-target effects, decreasing the specificity of the assays. To reduce off-target activity and enhance analytical performance, strategies such as improving crRNA design, engineering high-fidelity Cas variants, and optimizing reaction conditions are important [83]. (3) Lack of detection specificity and multiplexing capability: Respiratory infections are often co-infections that require multiplex detection. However, Cas12a and Cas13a can indiscriminately cut reporter molecules, causing signal cross-talk and false-positive readouts in complex reaction systems once activated [84]. Current approaches largely focus on improving crRNA design and engineering Cas proteins; however, systematic validation has been limited to clinically important respiratory pathogens. Multiplex detection using microfluidic platforms has been investigated. Still, problems with the complex design of primers and crRNAs, signal cross-talk, and the optimization of reporter systems remain to be solved for high-throughput, cost-effective testing without loss of analytical sensitivity [85]. RNA viruses—including SARS-CoV-2, influenza viruses, and respiratory syncytial virus (RSV)—are characterized by high mutation rates, which pose particular challenges for diagnostic assay design. To address this issue, one practical strategy is to design multiple crRNA panels that target conserved regions within the viral genome, especially in genomic segments prone to frequent variation. Such an approach can effectively offset the impact of target sequence drift caused by genetic mutations over time. Complementing this, the establishment of a dynamically updatable crRNA database, along with a flexible workflow that allows timely adjustment of crRNA designs, is essential to ensure the sustained diagnostic performance of CRISPR-based assays in the face of ongoing viral evolution [86]. (4) Clinical validation and standardization are not complete: CRISPR-based diagnostic technologies are still at an early stage, with a lack of standardization in the workflows and extensive clinical validation. Small sample sizes limit most studies, and there is a lack of large-scale, multi-center, randomized clinical trials to validate diagnostic accuracy, reliability, and reproducibility [87]. Inter-study comparability is further reduced by variability in the source of Cas enzymes, the design of the crRNAs, and the method of signal readout—fluorescence, colorimetric, or electrochemical. Currently, the clinical application of CRISPR/Cas detection technology remains in the exploratory stage, and the CRISPR/Cas respiratory pathogen detection platform reviewed in this article has not yet received routine in vitro diagnostic (IVD) approval [79]. Therefore, to facilitate the formal IVD approval of CRISPR/Cas detection systems in the future, it will be necessary to conduct large-scale, multicenter, randomized controlled clinical trials, establish standardized operating procedures and rigorous quality control systems, and undergo systematic regulatory review to verify their clinical safety, efficacy, and reliability [88].

To conclude, although there are still challenges to address, CRISPR/Cas-based systems show significant promise and broad application for detecting respiratory pathogens. Technological progress has made great strides in terms of feasibility and practicality with the use of engineered enzymes, integrated sample processing, and multiplex detection strategies. Further development of assay cost reduction and simplification of the assay workflow should be emphasized to enable their use in primary healthcare and resource-limited areas. The key approaches include optimizing CRISPR reaction systems, nucleic acid extraction steps, and developing freeze-dried reagents. The ability to integrate with microfluidic chips, isothermal amplification platforms, or portable and smartphone readout systems will enable the creation of fully integrated, hand-held point-of-care devices. These improvements will help diagnose pathogens quickly, enhance the judicious use of antimicrobials, and strengthen disease surveillance infrastructure, all of which will accelerate the clinical adoption of CRISPR/Cas diagnostic tools.

## Figures and Tables

**Table 2:** Comparison of Commonly Used Class II CRISPR/Cas Effector Proteins.

Cas Protein (Alias)	Target Nucleic Acid Type	Nuclease Domains	Guide RNA (gRNA)	Collateral Cleavage Activity
Cas9 (Csn1)	dsDNA	RuvC + HNH	crRNA + tracrRNA	None
Cas12a (Cpf1)	ssDNA, dsDNA	RuvC + Nuc	crRNA	ssDNA
Cas12b (C2c1)	ssDNA, dsDNA	RuvC	crRNA + tracrRNA	ssDNA
Cas13a(C2c2)	ssRNA	HEPN-1/HEPN-2	crRNA	ssRNA
Cas14a (Cas12f)	ssDNA/RNA	RuvC	crRNA + tracrRNA	ssDNA

**Table 3:** Comparison of CRISPR/Cas systems for respiratory pathogen detection.

Cas System	Applications	Advantages	Disadvantages
Cas9	MTB; MRSA	High specificity; well established; visual readout; portable	Requires pre-amplification; off-target effects
Cas12	SARS-CoV-2; HAdV;MTB; S.pneumoniae; VZV; M. pneumoniae; S. aureus	Robust trans-cleavage activity; high sensitivity; most widely applied	Signal crosstalk; PAM sequence dependency
Cas13a	SARS-CoV-2; Influenza virus (H1N1, H5N1); RSV	Direct RNA targeting; no reverse transcription needed	RNA targets prone to degradation; high mutation rate; RT step adds workflow time
Cas14a	DNA viruses (HAdV; VZV, etc.)	PAM-independent; high flexibility in target selection	Limited applications

## Data Availability

No new data were created or analyzed in this study. Data sharing is not applicable to this article.

## Abbreviations

The following abbreviations are used in this manuscript:

ARI	Acute respiratory infections
PCR	Polymerase chain reaction
LAMP	Loop-mediated isothermal amplification
RPA	Recombinase polymerase amplification
ERA	Enzymatic recombinase amplification
NNA	Non-nucleic acid
dsDNA	Double-stranded DNA
sgRNA	Single-guide RNA
ssDNA	Single-stranded DNA
PAM	Protospacer adjacent motif
DSB	Double-strand break
SARS-CoV-2	Severe Acute Respiratory Syndrome Coronavirus 2
HCR	Hybridization chain reaction
3D-SDNC	Three-dimensional spherical DNA nanostructure
gFETs	Graphene field-effect transistors
CPA	Cross-priming amplification
QDNB	Quantum dot nanobead
MTB	Mycobacterium tuberculosis
SP	Streptococcus pneumoniae
MP	Mycoplasma pneumoniae
MCDA	Multiple cross-displacement amplification
MRSA	Methicillin-resistant Staphylococcus aureus
ECL	Electrochemiluminescence
AIDS	Acquired immunodeficiency syndrome
LFA	Lateral flow assay
HPAI	Highly pathogenic avian influenza
